# The variation in crown-root morphology of anterior teeth assessed with cone-beam computed tomography

**DOI:** 10.1590/2177-6709.27.1.e222079.oar

**Published:** 2022-05-06

**Authors:** Xiao-ming WANG, Ling-zhi MA, Mei-fang YAN, Jun ZHENG, Mi WANG, Xue HUI

**Affiliations:** 1 Lanzhou University, School of stomatology (Lanzhou, China).; 2 Stomatological Hospital of Kunming Medical University, Department of Orthodontics (Kunming, China).; 3 The Affiliated Suzhou Hospital of Nanjing Medical University, Department of Stomatology (Suzhou, Jiangsu Province, China).; 4 Myour Dental Clinic (Suzhou, Jiangsu, China).

**Keywords:** Anterior teeth, Crown-root morphology, Collum angle, Labial surface angle, Cone-beam CT

## Abstract

**Objective:**

To determine the discrepancy of crown-root morphology of anterior teeth, using cone-beam computed tomography (CBCT), and to provide a guidance for proper torque expression.

**Methods:**

A total of eligible 200 CBCT were imported into Invivo v. 5.4 software, to obtain the middle labio-lingual sections of anterior teeth. AutoCAD 2007 software was applied to measure the crown-root angulation (Collum angle) and the angle formed by a tangent to the center of the labial surface and the long axis of the crown (labial surface angle). SPSS 18.0 was used for statistical comparisons of the two measurements, at the level of *p*< 0.05, and the Pearson correlation analysis was applied to investigate the association between the two measurements.

**Results:**

The value of Collum angle in maxillary central incisor was close to 0°. Significantly negative Collum angle in lateral incisors and maxillary canine, and positive value in mandibular canine were detected (*p* < 0.001). The labial surface angle in canine was significantly greater than the intra-arch incisors (*p*< 0.001), and no significant difference was detected between the central and lateral incisors (*p* > 0.05). Notably, there was also a significant positive correlation between the two measurements.

**Conclusions:**

The crown-root angulations were greatly different among anterior teeth. Accompanying the obvious crown-root angulations, the canines both in maxillary and mandibular arches presented considerable labial surface curvatures. Hence, equivalent deviation during bracket bonding might cause greater torque expression error and increase the risk of alveolar fenestration and dehiscence.

## RESUMO

**Objetivo:**

Determinar a discrepância na morfologia coroa-raiz de dentes anteriores, utilizando tomografia computadorizada de feixe cônico (TCFC), e fornecer parâmetros para a expressão apropriada do torque.

**Método:**

No total, 200 tomografias elegíveis foram importadas para o *software* Invivo 5.4 para obtenção das secções médias vestibulolinguais dos dentes anteriores. O*software* AutoCAD 2007 foi usado para medir a angulação coroa-raiz (ângulo Collum) e o ângulo formado por uma tangente ao centro da superfície vestibular da coroa e o longo eixo da coroa (ângulo da superfície vestibular). O *software* SPSS 18.0 foi utilizado para as comparações estatísticas das duas medições, com nível de significância de *p*< 0,05, e a análise de correlação de Pearson foi aplicada para investigar a associação entre as duas medições.

**Resultados:**

O valor do ângulo Collum do incisivo central superior foi próximo a 0°. Foram detectados valores significativamente negativos para o ângulo Collum nos incisivos laterais e caninos superiores, mas valores positivos nos caninos inferiores (*p*< 0,001). O ângulo da superfície vestibular no canino foi significativamente maior do que nos incisivos intra-arcada (*p*< 0,001), e nenhuma diferença significativa foi detectada entre incisivos centrais e laterais (*p*> 0,05). Também foi observada uma correlação positiva significativa entre as duas medições.

**Conclusões:**

As angulações coroa-raiz foram muito diferentes entre os dentes anteriores. Os caninos superiores e inferiores apresentaram considerável curvatura na superfície vestibular, associada a uma evidente angulação coroa-raiz. Consequentemente, desvios durante a colagem de braquetes podem desencadear maior erro na expressão de torque e aumentar o risco de fenestração alveolar e deiscência, sendo necessária uma avaliação antes da colagem.

## INTRODUCTION

Appropriate anterior torque expression is essential for establishing stable occlusion and satisfying esthetics. In the past two decades, researchers have focused at the influence of the morphology of anterior alveolar height and thickness on anterior torque,^1^ while the tooth morphological variation has been frequently ignored. In 1984, Bryant et al^2^ firstly noticed the variability in the maxillary permanent central incisor morphology by establishing three anatomic features, and investigated the discrepancies among different Angle malocclusions. Thereafter, two features were widely adopted by the following studies.^3^ ^-^ ^6^

**Figure 1:**
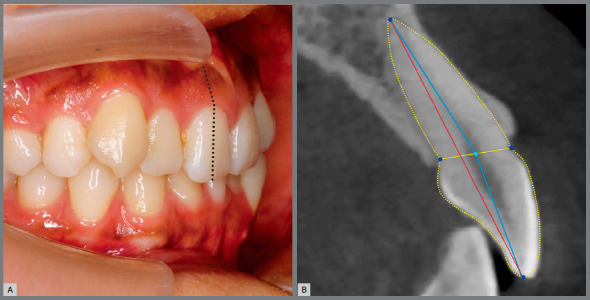
A) The intraoral photo potentially indicated the crown-root angulation phenomenon for the inclined root and upright crown in maxillary central incisor. B) Further lateral radiography confirmed that the long axes of crown and root didn’t coincide with each other, or the connection between incisor edge and root apex (red line) didn’t pass through the mid-point of the labial and lingual cementoenamel junction (yellow line).

One feature was the crown-root angulation (Collum angle, CA) formed by the long axis of root and crown, in the labiolingual direction. Previous studies found the connection between root apex and incisal edge did not pass through the middle point of the connection between labial and lingual CEJ^2^ (Fig 1), which further restricted the degree to which the roots of these teeth can be lingually torqued when close to the maxillary palatine cortical bone plate.^2^ ^,^ ^7^ ^,^ ^8^ Our recent study concluded that the maxillary central incisor in patients with sagittal skeletal Class II malocclusion and mandibular incisor with Class III malocclusion presented remarkable crown-root angulation.^6^ Hence, the diversity in CA might result in unmanageable root position, increasing the incidence of dehiscence and fenestration, and limiting normal torque expression.^1^ ^,^ ^3^ ^,^ ^9^ ^,^ ^10^ However, little was known about the values of CA and the detailed differences among the anterior teeth.

The other feature was the labial surface angle (LSA, representing the facial contour of anterior teeth), which was formed by a tangent to the bracket site on the labial surface and the long axis of the crown, from a proximal view.^2^ The significant amount of variation in LSA potentially affected the precision of torque expression and facio-lingual inclination.^5^ ^,^ ^6^ A previous study found that the LSA of maxillary canines was significantly different at the same location of different canines.^11^ Moreover, the LSA of 198 maxillary central incisors (extracted teeth and teeth traced from the cephalometric radiograms) ranged from 7° to 24°.^2^ Thus, the preoperative assessment of individual LSA might be essential for appropriate brackets selection and later torque adjustment. However, the systematic comparison of the facial contour of maxillary and mandibular anterior teeth is still necessary.

Previous studies of CA and LSA of maxillary central incisor used cephalometric radiographs, thus presenting the disadvantages of poor accuracy in two-dimensional images. Currently, CBCT is widely used in oral examinations, with the advantage of multiplanar, thin-sliced images that are not impaired by superimposition,^12^ ^,^ ^13^ but its application in crown-root morphological study remains incipient.^6^ Moreover, according to previous studies, the two morphological measurements in central incisors were highly associated with the variation of torque,^6^ but the characteristics of other anterior teeth are still unknown. On this basis, the present article sought to investigate the variations in the crown-root morphology (CA and LSA) of maxillary and mandibular central and lateral incisors, and canines using CBCT data. Invivo v. 5.4 software was used to capture images, and the analysis were performed with AutoCAD. Furthermore, the regularity of crown-root morphology and effect on torque expression among different anterior teeth were verified. 

## MATERIAL AND METHODS

### STUDY DESIGN

The design was a cross-sectional and retrospective study using dental records. All the participants had CBCTs taken for clinical orthodontic needs, and approved its use for clinical research. Firstly, a power analysis established by G*Power (version 3.1.9.4, Franz Faul, Universität Kiel, Kiel, Germany) software, based on 1:1 ratio between groups, with sample size of 200 eligible cases, would give more than 80% power to detect significant differences, with 0.17 effect size and at the α= 0.05 significance level.

### SAMPLE SELECTION AND CLASSIFICATION

This study was undertaken with the CBCT scans selected from the archives of the Department of Stomatology, the Affiliated Suzhou Hospital of Nanjing Medical University. By April 2019, 3,419 sets of images were stored in the database of the department. The experiments were carried out in strict accordance with the guide for patient at Suzhou Municipal Hospital, and were approved by the Committee on the Ethics of Human Experiments (K2016051).

Initially, 452 CBCT images of patients were selected according to the criteria presented in Table 1. CBCT data was established using the Implagraphy (Vatech Co., Yongin, Korea), with a visual range of 20 × 19cm^2^, tube voltage of 90 kV, tube current of 3.5mA, slice thickness of 0.20 mm, exposure time of 24s. During scanning, patients were instructed to keep the interpupillary line and Frankfurt plane parallel to the ground, making the facial midline consistent with the median reference line of the machine. They were asked to keep central occlusion and to not swallow.^6^

**Table 1:** Sample selecting criteria.

Inclusion criteria	Exclusion criteria
Permanent dentition, completely developed root, no apparent bending and no resorption	Anterior root with periapical lesions or apparent bending, containing supernumerary teeth embedded in alveolar bone
Intact contour of the crown, and no apparent abrasion	Crown with obvious abrasion
Mild crowding, and no apparent rotation in anterior teeth	Moderate to severe crowding, or obvious rotation in anterior teeth
No caries, filling, restoration history or periodontitis in anterior teeth	Caries, filling, restorative treatment, or periodontitis leading to unstable anterior teeth
No orthodontic, functional orthopedic treatment, cleft lip palate, or orthognathic surgery history	With orthodontic, functional orthopedic treatment, cleft lip palate, or orthognathic surgery history
No oral deleterious habit, occlusion interference, swallowing and respiratory disorder, and facial or spinal abnormalities	With oral deleterious habit and the mandible located in dysfunctional or unstable position, or jaw cyst, cancer, injury and abnormalities
Sagittal skeletal Class I malocclusion and normal vertical growth pattern	Sagittal skeletal Class II or III malocclusion, hypodivergent or hyperdivergent vertical growth pattern
Clear CBCT image	Blurred CBCT image

Further, the intraoral photographs were used to select the Angle Class I types. The Invivo v. 5.4 software was used to capture the lateral cephalometric radiographs, which were then imported into Dolphin v. 11.0 for cephalometric analysis. Finally, a total of 200 individuals proved to be eligible (skeletal Class I and average vertical growth pattern). The detailed criteria and average information of subjects are shown in Table 2.^6^ ^,^ ^14^

**Table 2:** Information of the sample.

Attributions	Average values or distribution
Age	26.7 ± 8.2 years (From 18 to 40)
Sex	Male, 66 (Female, 134)
Race	Han nationality
ANB (1° ≤ ANB ≤ 5°)	3.7 ± 1.1°
Wits (−3.6 mm ≤ Wits ≤ 0.7 mm)	-1.5 ± 2.1mm
SN-MP (27° ≤ SN-MP ≤ 37°)	32.8 ± 4.0°
FHI (S-Go/N-Me) ( 62% ≤ FHI ≤ 68%)	65.4 ± 2.5%

### MEASURING THE CAPTURED IMAGE

**Figure 2:**
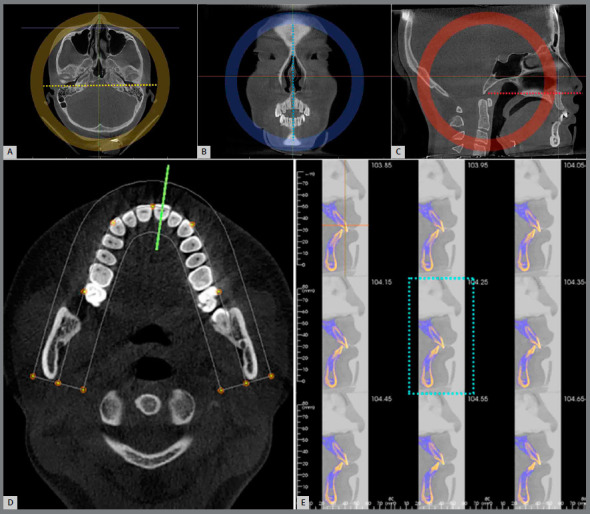
Measuring image capture: the natural position of the head is adjusted in three dimensions. **A**) Horizontal view, with the connection of bilateral Porions parallel to the horizontal reference line. **B**) Coronal view, with vertical reference line passing through the median Nasion. **C**) Sagittal view, with horizontal reference line passing through the palatal plane. **D**) The bunch of cutting lines (green) was vertical to the labial surface of crown and located centrally. **E**) The median sagittal views were established with nine layers, interval of 0.10 mm, and the middle one was the measuring image (blue dotted frame).

The CBCT data were three-dimensionally adjusted using Invivo v. 5.4 software (Anatomage Dental) to orient the head in three dimensions, as previously reported.^6^ ^,^ ^15^ Briefly, the horizontal plane was rotated to pass through the horizontal reference line, connecting the upper margins of bilateral Porion (Fig 2A, yellow dotted line); then, the coronal plane passing through bilateral Porion was rotated to make the perpendicular reference line via Nasion (Fig 2B, blue dotted line); lastly, the sagittal plane was tilted clockwise or counterclockwise until the palatal plane (defined by the anterior nasal spine and the posterior nasal spine) was set parallel to the horizontal reference line (Fig 2C, red dotted line). For the interception of the measuring image, the labio-lingual sections of anterior teeth were adjusted to capture, using the Arch Section tab. It was essential that the bunch of cutting lines (green) was set vertical to the connection between mesial and distal edges of crown, and located at the center in horizontal view (Fig 2D). Thus, the median of the nine images would be selected as the measuring one (Fig 2E).

### MARKER AND MEASUREMENT

**Figure 3:**
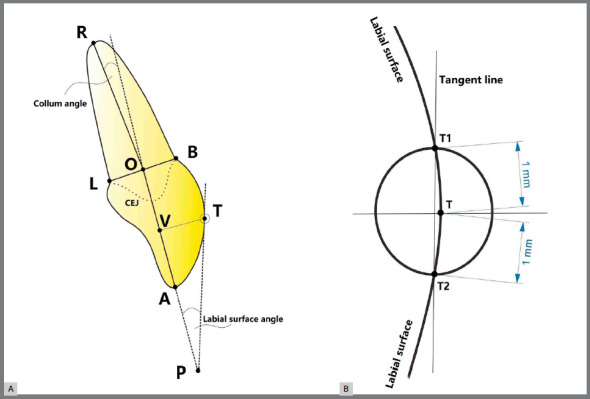
A) The Collum angle is formed by the extension of the long axis of the crown and the long axis of the root. B) The labial surface angle is formed by Tangent L passing through upper and lower intersections on labial surface of crown formed by a circle with the center at T and 1-mm radius.

AutoCAD (Autodesk, San Rafael, CA) software was used as previously described, with little modifications, to measure the values of CA and LSA.^5^ ^,^ ^6^ Point A was marked at the incisor edge and point R, at the root apex. CEJ was set at the labial or lingual cementoenamel junction. Points B and L were marked at the labial and lingual cementoenamel junctions, respectively. Point O was defined at the midpoint between the above two points. The lines AO and RO represented the long axis of the crown and root, respectively. Point T was set as the tangent point on the labial surface of the crown, which was the intersection of the perpendicular line of AO and the labial surface of the crown with the point V (Fig 3A). The tangent line via point T was drawn approximately by the line passing through points T1 and T2, which were the intersections of a circle with the point T center and 1-mm radius on the labial surface of the crown (Fig 3B). 

Thus, the Collum angle (CA) was formed by the line RO and reverse extension line of AO. When line RO located lingual side to the extension line, the CA was defined as a positive value; otherwise, the labial side was defined as negative, and the coincidence was zero. Labial surface angle (LSA) was formed by the tangent line and forward extension line of AO, with point P as the vertex (Fig3A).

### STATISTICAL ANALYSIS

Statistical analysis was carried out using the SPSS software (version 13.0, SPSS, Chicago). Firstly, the Kolmogorov-Smirnov test was used to test the normality of the distribution for the value of CA and LSA in each group, respectively. Levene’s variance homogeneity test was used to test the homogeneity of variance among groups, both of which were proved to be normally distributed, with homogeneity of variance among groups. Further statistical comparisons of CA and LSA in different groups were performed by one-way analysis of variance (ANOVA) and Scheffe test. The Pearson correlation analysis was applied to investigate the association between CA and LSA in the same tooth (‘r’ was the Pearson correlation coefficient). The level of statistical significance was set at *p*< 0.05(*), *p* < 0.01(**), and *p*< 0.001(***).

### METHOD ERROR

To assess the intra-observer and inter-observers error, repeated measurements performed on all the samples were measured by two operators on two occasions, with a two-week interval, and analyzed with Student’s *t*-test for paired samples, adopting an α-level of 0.05. The technical error of measurement (TEM) was assessed with the Dahlberg’s formula:^6^ ^,^ ^16^

TEM=√∑d/2n

in which d_i_ was the difference between the first and second measurement on the i^th^ sample, and n was the whole sample number. As a result, the intra-observer errors were 0.27° for CA and 0.39° for LSA, and inter-observers error were 0.47° for CA and 0.52° for LSA. The values indicated that the analysis was reliable, since all the measurements presented no significant difference, according to the *t*-test (*p* > 0.05). 

## RESULTS

 1. Comparison of CA among different types of intra and inter-dental arch teeth (Tables 3 and 4) 

**Table 3:** CA / LSA of different maxillary and mandibular teeth, using one-way ANOVA (degrees).

	U1	U2	U3	L1	L2	L3	F	ANOVA *p*-value
	Mean ± SD	Mean ± SD	Mean ± SD	Mean ± SD	Mean ± SD	Mean ± SD		
CA	0.17 ± 5.11	-5.67 ± 5.74	-5.56 ± 4.63	-3.97 ± 4.49	-6.50 ± 4.03	3.70 ± 4.91	137.92	0.000
LSA	15.51 ± 2.91	15.92 ± 3.50	20.07 ± 3.66	14.40 ± 3.20	14.76 ± 3.25	18.27 ± 3.07	91.02	0.000

CA = Collum angle; LSA = Labial surface angle; SD = Standard deviation; U1 = Maxillary central incisor; U2 = Maxillary lateral incisor; U3 = Maxillary canine; L1 = Mandibular central incisor; L2 = Mandibular lateral incisor; L3 = Mandibular canine.

**Table 4:** The difference value of CA / LSA between different types of anterior teeth, using Scheffe test (degrees).

	U1-U2		U1-U3			U2-U3		L1-L2		L1-L3		L2-L3		U1-L1		U2-L2		U3-L3	
	Mean	*p*	Mean	*p*	Mean	*p*	Mean	*p*	Mean	*p*	Mean	*p*	Mean	*p*	Mean	*p*	Mean	*p*
CA	5.85	0.00	5.73	0.00	-0.12	1.00	2.58	0.00	-7.63	0.00	-10.20	0.00	4.10	0.00	0.83	0.72	-9.26	0.00
LSA	-0.42	0.20	-4.55	0.00	-4.14	0.00	-0.37	0.25	-3.87	0.00	-3.51	0.00	1.12	0.04	1.17	0.03	1.80	0.00

CA = Collum angle; LSA = Labial surface angle; U1 = Maxillary central incisor; U2 = Maxillary lateral incisor; U3 = Maxillary canine; L1 = Mandibular central incisor; L2 = Mandibular lateral incisor; L3 = Mandibular canine.

**Figure 4:**
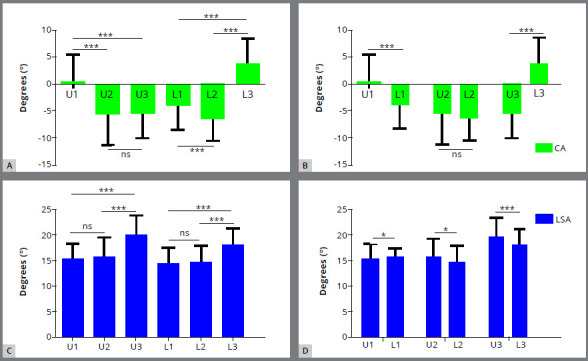
Distribution of CA and LSA in maxillary and mandibular anterior teeth. CA = Collum angle; LSA = Labial surface angle; SD = Standard deviation; U1 = Maxillary central incisor; U2 = Maxillary lateral incisor; U3 = Maxillary canine; L1 = Mandibular central incisor; L2 = Mandibular lateral incisor; L3 = Mandibular canine.

In maxilla, the mean values of CA in central incisor approached to 0° (0.17 ± 5.11°). The magnitude was significantly less than lateral incisor (-5.67 ± 5.74°, *p*= 0) and canine (-5.56 ± 4.63°,*p*= 0), indicating less identical crown-root angulation. The CAs in lateral incisor and canine were considerably negative, indicating that the long axis of root deviated labially from the direction of the long axis of crown. However, there was no significant difference between the lateral incisor and canine (*p*= 1.000) (Fig 4A). In mandible, the mean values of CA in central incisor (-3.97 ± 4.49°) and lateral incisor(-6.50 ± 4.03°) were negative and presented significant discrepancy between each other (*p*= 0), while CA in canine was significantly positive (3.70 ± 4.91°, *p*= 0). Thus, the long axis of crown deviated labially from the direction of root in mandibular central and lateral incisors, while deviated lingually in canine (Fig 4A). Comparing the values between the same types of interdental arch teeth, significant discrepancies were observed between upper and lower central incisors (*p*= 0), and similar result was also detected between canines (*p*= 0). However, no significant discrepancy in the value of CAs between upper and lower lateral incisors were found (*p*= 0.72) (Fig 4B). 

2. Comparison of LSA among different types of intra and inter-dental arch teeth (Tables 3 and 4) 

In maxilla, the mean values of LSA for canine presented significantly greater (20.07 ± 3.66°) than for central (15.51 ± 2.91°) and lateral incisors (15.92 ± 3.50°), suggesting a greater facial curvature of crown. No significant statistical difference was detected between the central and lateral upper incisors (*p*= 0.20) (Fig 4C). In mandible, the results distribution was similar to maxillary distribution. The mean value of LSA in canine (18.27 ± 3.07°, *p*= 0) was also significantly greater than central (14.40 ± 3.20°, *p*= 0) and lateral incisors (14.76 ± 3.25°, *p*= 0), and no significant difference between the central and lateral lower incisors was found (*p*= 0.25) (Fig 4C). Further comparison of the values between same types of inter-dental arch teeth demonstrated that the all of the three LSAs in upper teeth appeared greater than for the same types of opposite teeth (*p*= 0.04, *p*= 0.03, *p*= 0), indicating a greater labial surface curvature in maxillary anterior teeth (Fig 4D).

3. Pearson correlation test between the CA and LSA within the same tooth (Table 5)

**Table 5:** Pearson correlation analysis detects the association between CA and LSA.

Anterior tooth	CA-LSA	
	r	P
U1	-0.101	0.078
U2	0.190	0.004
U3	0.230	0.001
L1	0.219	0.001
L2	0.362	0.000
L3	0.140	0.025

CA = Collum angle; LSA = Labial surface angle; SD = Standard deviation; U1 = Maxillary central incisor; U2 = Maxillary lateral incisor; U3 = Maxillary canine; L1 = Mandibular central incisor; L2 = Mandibular lateral incisor; L3 = Mandibular canine.

**Figure 5:**
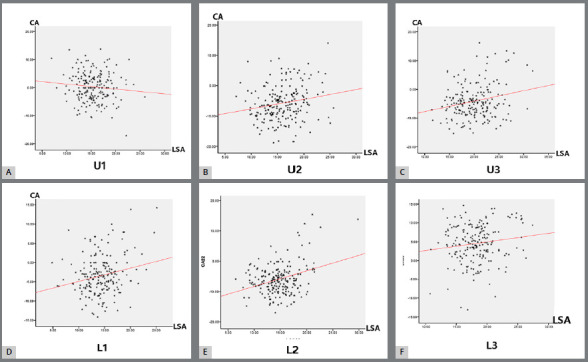
The CA and LSA were significantly and positively correlated in anterior teeth (**B-E**), except for the maxillary central incisor (A). CA = Collum angle; LSA = Labial surface angle; SD = Standard deviation; U1 = Maxillary central incisor; U2 = Maxillary lateral incisor; U3 = Maxillary canine; L1 = Mandibular central incisor; L2 = Mandibular lateral incisor; L3 = Mandibular canine.

The consistency of the difference distribution suggested that there might be some extent of correlation between the two measurements within the same tooth. Thus, the association between CA and LSA within the same tooth was verified using the data from all the samples. As a result, except for the maxillary central incisor, where there was no significant correlation between the two measurements (r = -0.101, *p*= 0.078), the Pearson correlation test indicated that the two measurements were significantly and positively correlated in other anterior teeth, even though the correlation coefficient was relatively weak (Fig 5). Together, the meaningful positive correlation suggested a regular effect on torque limitation, which was analyzed in the following discussion.

## DISCUSSION

The preadjusted, or straight-wire, appliance is widely accepted by orthodontics, based on the assumption that the morphologies of teeth crowns among the individuals present discrete variation. Thus, without considering the variation in facial surface contours, orthodontists believe that the bracket preadjusted torque promotes a coincident extent of labio-lingual inclination with the same setting.^6^ ^,^ ^17^ However, researchers found that the facial curvatures of teeth were not identical between individuals or in different heights of bracket placement.^5^ ^,^ ^17^ Some orthodontists demonstrated that the variations in such tooth morphology could be more important than the variations between the different types of preadjusted brackets.^10^ Recently, Kong et al^5^ determined the LSAs in 77 incisors at a height of 3.5-5.0 mm gingivally from the incisal edge, and found that the variable extents were significantly greater than the variations between different types of preadjusted appliances. Using the lateral cephalometric radiographs, researchers found that the crown-root angulation formed by the long axis of the crown and root of maxillary central incisor was significantly greater in Angle Class II division 2 malocclusion than other types, which would increase the danger of perforating the palatal cortical plate, resulting in different root positions with constant crown positions.^3^ ^,^ ^10^ ^,^ ^18^ Our study further confirmed that the maxillary incisor in sagittal skeletal Class II malocclusion and mandibular incisor in Class III malocclusion present obvious crown-root angulation,^6^ which importantly influenced the normal distribution of strains in the periodontal ligament after torque application.^4^ ^,^ ^8^ Thus, it seems that the two morphological features are essential to safely apply torque bend. A large number of relevant studies focused on the maxillary central incisor, including our previous study;^6^ moreover, the researches were primarily conducted on cephalometric radiographs, with the disadvantage of magnifying distortion and rough manual tracing. Hence, a systematic evaluation on all the anterior teeth using CBCT is necessary. 

In the present study, the average absolute value of CA in maxillary central incisor nearly approached to 0° (0.17°), which was smaller than the former measurements using the 77 extracted teeth (0.88°)^5^ or 200 lateral skull radiographs (-0.7°).^19^ A previous study^6^ found that it was significantly greater in sagittal skeletal Class II malocclusion (5.18°) than other malocclusion types, and not excluding the Class II might be the reason for the larger results in previous studies. The maxillary lateral incisor and canine presented significant negative CAs, compared with the central incisor, suggesting that the roots were relatively bent toward the labial cortex, and the value of canine was similar to previous results.^5^ The negative CA in maxillary canine was also detected by Germane et al^17^ and Kong et al.^5^ Thus, this might be one of the important reasons for the frequent dehiscence or fenestration when the maxillary canine is retracted. Regarding the CA in lateral incisor, we found a negative value and no statistical discrepancy, comparing with the maxillary canine, which was consistent with other study.^5^ The maxillary lateral incisors are the teeth with the greatest morphological variability after the third molars, with the prevalence of peg-shaped teeth ranging from 0.6% to 9.9%, due to the special genetic and surrounding environmental factors.^20^ ^,^ ^21^ Regarding the CA in mandible, the values in central and lateral incisors were significantly negative, also indicating the labial root bent and increasing risk of dehiscence and fenestration on labial cortex. Consistent with the former researches,^5^ ^,^ ^17^ the canine presented a significantly positive result in our study, indicating a lingual root bent relative to crown. Nevertheless, alveolar dehiscence or fenestration was thought to easily occur in the mandibular canine because of the thin alveolar plate.^22^ Moreover, when comparing the same types of inter-dental arch teeth, we also found significantly different crown-root angulations among the anterior teeth. Just as Feres et al^23^ and our previous study^6^ suggested, the estimation of CA was essential for better tooth movement planning, especially regarding to root palatal torqueing. 

**Figure 6:**
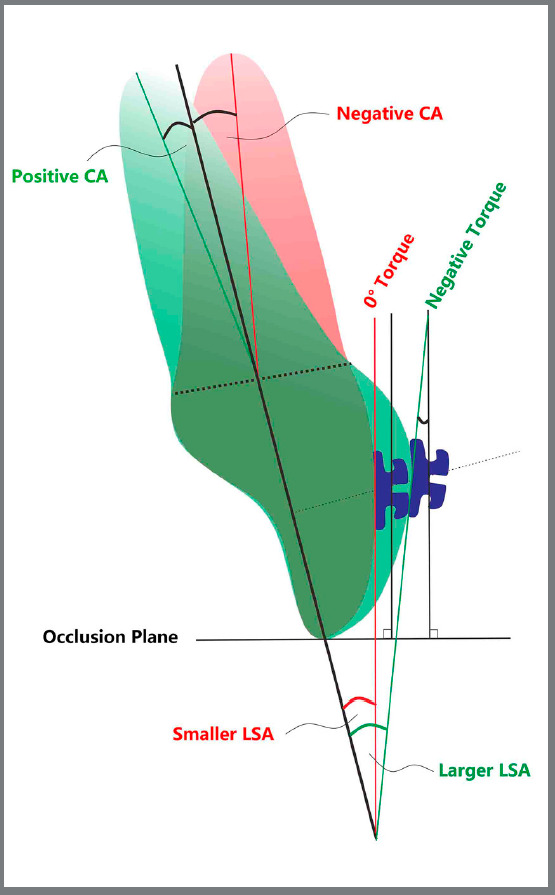
Compared with the negative Collum angle (root bent labially), the positive Collum angle (root bent lingually) associated with larger labial surface angle, which resulted in increased labial surface curve and negative torque.

LSA stands for the labial surface angle of the crown, and proved to be another anatomical feature of the tooth. Germane et al^17^, measuring 50 of each type of tooth, found that the standard deviations of LSA increased from the central incisor to the first molar in both the maxilla and mandible, indicating greater labial surface curvature variation when moving posteriorly. In the same tooth, at different heights from incisal edge, each 0.5-mm increase leads to a torque reduction of about 2°.^5^ It was demonstrated that the significant variation of LSA would cause a wide range of torque (from 12.3° to 24.9°) when measuring at 4.5 mm from the incisal edge.^9^ In the present study, we integrated all the anterior teeth and determined the LSAs formed at the uniform bonding sites of straight-wire brackets. We found that both in maxilla and mandible, the LSA of canine was significantly greater than the intra-dental arch central and lateral incisors^5^, indicating that equivalent deviation during bracket bonding might cause greater torque expression error in canine than in incisors. Moreover, no significant discrepancy was found between the intra-dental arch central and lateral incisors, supporting the rationality of similar bracket base shape of central and lateral incisors^24^. Between the mandibular central and lateral incisors, we detected no significant discrepancy in LSA, coinciding with Germane’s et al.^17^ results, where they got slight changes (within 1°) at different height of potential bracket positions on crown. Thus, the similar facial surface contour between mandibular central and lateral incisors further supported that the coincident torqueing design for the two incisors was reasonable^25^. In addition, comparing with the LSA of same types of tooth between the dental arches, the maxillary teeth were generally greater, indicating that equivalent deviation during bracket bonding might cause greater torque expression error in maxilla. Interestingly, like our previous study^6^, we also detected a significant positive correlation between the value of CA and LSA, meaning that the labial surface curvature was correspondingly greater in cases with remarkable crown-root angulation (Fig 6).

The cause of variable crown-root morphology is still debatable. Although developmental biologists believe the genetic determinants in the tooth morphological development,^26^ the majority of orthodontists proposed that the perioral muscle force plays an important role in the formation of crown-root angulation and variable labial surface curvature.^7^ ^,^ ^17^ ^,^ ^27^ During the eruption and occlusion establishing stage of anterior teeth, the teeth are actively extruded by the forces from tongue and opposite teeth labially, and the force from lip lingually.^6^ ^,^ ^22^ Thus, based on a previous study, we suppose that the position of tongue and lip relative to crown and the extent of anterior overbite may be regularly associate with the variable crown-root morphology. Therefore, further research is necessary to verify the potential relationship.

However, it is worth highlighting that the present study mainly focused on the sagittal variation of tooth morphology, but the variation and stress analysis of tooth in three-dimension during torqueing will be recommend to more reasonably explain the effect on the periodontal ligament and alveolar bone. Moreover, the movement restriction of root can be caused by the poor alveolar bone, which should also be integrated into torqueing.

## CONCLUSION

There was great variability in the crown-root morphology of anterior teeth. The maxillary central incisor presented the minimum crown-root angulation, while the lateral incisor proved to be the most variable. Compared with the incisors, the root of canine obviously bent toward the labial cortex in maxilla, while lingual in mandible. Accompanied by the remarkable crown-root angulation, the canines both in maxilla and mandible formed greater labial surface curvature of crown than incisors. Thus, equivalent deviation during bracket bonding might cause greater torque expression error. Together, in addition to the estimation of local alveolar height and thickness, more attention should be given to the crown-root morphologies of anterior teeth before torqueing, to prevent alveolar fenestration, dehiscence and root absorption.
